# Modulating inflammation and oxidative stress in rheumatoid arthritis: a systematic review of nutraceutical interventions

**DOI:** 10.1007/s10787-025-01976-8

**Published:** 2025-09-30

**Authors:** Camila Leiva-Castro, Ana M. Múnera-Rodríguez, Gádor Torres-Joya, Francisca Palomares-Jerez, Soledad López-Enríquez

**Affiliations:** 1 Department of Medical Biochemistry and Molecular Biology and Immunology, School of Medicine, University of Seville, Av. Sanchez Pizjuan S/N, 41009 Seville, Spain; 2 Institute of Biomedicine of Seville (IBiS)/Virgen del Rocío University HospitalVirgen Macarena University HospitalUniversity of Seville/CSIC, Seville, Spain

**Keywords:** Rheumatoid arthritis, Nutraceuticals, Inflammation, Oxidative stress, Alternative therapies, Immune modulation, Autoimmune disease

## Abstract

Rheumatoid arthritis is a chronic autoimmune disease characterized by persistent synovial inflammation and progressive joint destruction. The gut microbiome has emerged as a key factor in the regulation of the immune system, and its dysbiosis has been implicated in the pathogenesis of rheumatoid arthritis. Nutraceuticals, including probiotics, omega-3 fatty acids, coenzyme Q10, vitamin D, and polyphenols, have shown potential in modulating the gut microbiota and inflammatory pathways. This review explores the interplay between nutraceuticals, gut microbiota, and immune function in rheumatoid arthritis, with attention to pharmacokinetics and safety. We discuss recent clinical evidence, elucidate molecular mechanisms of action, and highlight future research directions for integrating nutraceuticals into therapeutic strategies for rheumatoid arthritis.

## Introduction

Rheumatoid arthritis (RA) is a chronic autoimmune disorder with a global prevalence ranging from 0.3 to 1.2%, with women accounting for approximately two-thirds of affected individuals. This disease significantly impairs patient’s quality of life and is associated with substantial social and healthcare burdens (Miguel-Lavariega et al. 2023). It is characterized by inflammation of the synovial membrane, resulting in pain, swelling, and progressive joint destruction in affected individuals (Smith and Bermam, 2022). RA is a multifactorial disease in which genetic, environmental, and lifestyle factors contribute to its pathogenesis; however, the precise combination of triggers remains unclear. Notably, genetic predispositions, such as HLA-DR4 and HLA-DRB1, alongside environmental exposures including smoking and infections, are known to play a significant role in disease onset (Alwarith et al. 2019).

Once triggered, the immune system becomes central to the disease’s progression. During the autoimmune response, T and B lymphocytes are activated and migrate to the synovial membrane, leading to a highly inflammatory microenvironment. This is marked by elevated levels of pro-inflammatory cytokines, such as tumor necrosis factor-alpha (TNF-α), interleukin (IL)-1, IL-6, and metabolites of arachidonic acid (Alwarith et al. 2019). Despite the critical advancements in RA treatment strategies, effective management remains challenging for a significant subset of patients, particularly in terms of achieving sustained remission and preventing irreversible joint damage.

### Immunopathogenesis of rheumatoid arthritis

RA origins from a complex interplay between genetic susceptibility and environmental factors, culminating in a dysregulated immune response that drives chronic inflammation within the joints. Both the innate and adaptive immune systems are involved in disease pathogenesis, with T and B cells playing key roles in the initiation and maintenance of inflammation. These immune cells infiltrate the synovial tissue, where they interact with resident macrophages and fibroblasts, triggering the secretion of pro-inflammatory cytokines and tissue-degrading enzymes, including TNF-α, IL-1, and IL-6. These mediators activate downstream signaling pathways, ultimately resulting in tissue destruction and synovial hyperplasia (Ding et al. 2023).

**Fig. 1:**
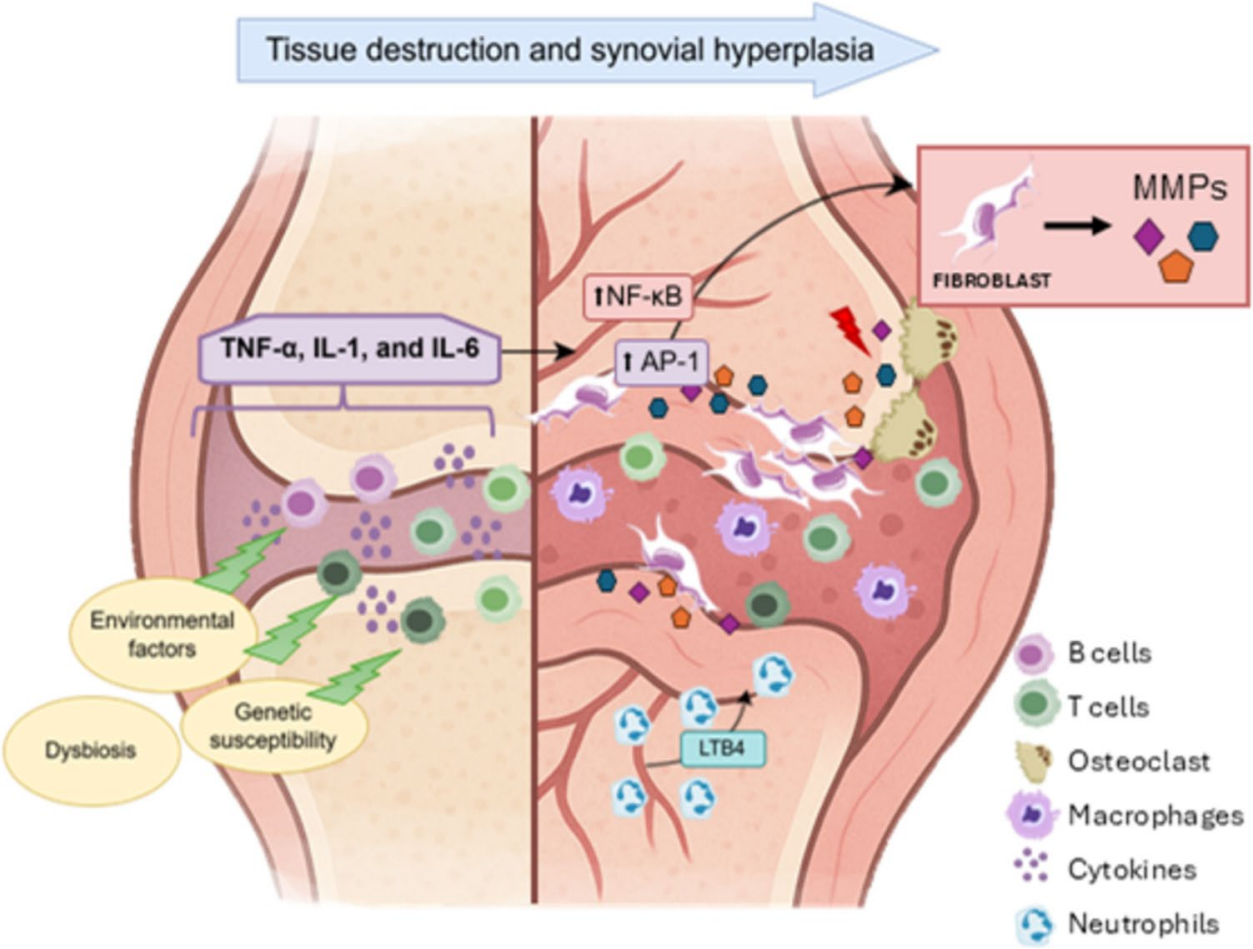
Schematic representation of the main immune cells and signaling pathways involved in the immunopathogenesis of RA. On the left, environmental triggers, genetic predisposition, and gut dysbiosis initiate an inflammatory cascade characterized by the release of pro inflammatory cytokines such as TNF-α, IL-1, and IL-6. These cytokines activate immune cells including B cells, T cells, macrophages, neutrophils, and osteoclasts. On the right, fibroblasts respond to these signals by activating the NF-κB and AP-1 pathways, leading to the production of matrix metalloproteinases (MMPs), which degrade extracellular matrix components and contribute to joint damage

A hallmark feature of RA is the formation of pannus, a hypertrophic synovial tissue that aggressively invades cartilage and bone. Pannus tissue is enriched with matrix metalloproteinases (MMPs), which are responsible for degrading the extracellular matrix and contribute to structural damage (Miller et al. 2009; Burrage et al. 2006). Key transcription factors, such as nuclear factor-kappa B (NF-κB) and activator protein 1 (AP-1), regulate the expression of these enzymes, contributing to the pathological remodeling of joint tissues (Ding et al. 2023; Burrage et al. 2006), (Fig. 1).

Therapeutically, RA management focuses on controlling inflammation, alleviating symptoms, and preventing joint damage. In this sense, the “treat to target” approach has been a significant advancement in the treatment of RA. This strategy aims to achieve early remission or, failing that, to minimize inflammatory activity through personalized, intensive treatment that is quickly adjusted based on the patient’s clinical response. First-line therapy typically includes nonsteroidal anti-inflammatory drugs (NSAIDs), corticosteroids, and methotrexate. However, when this treatment is unable to achieve an ideal outcome, it is recommended to incorporate another conventional disease-modifying antirheumatic drug (DMARD), opt for biological therapies (targeting TNF-α, IL-6, CD20, IL-1, and T lymphocytes), or use Janus kinase (JAK) inhibitors (George et al. 2020).

These therapies are designed to counteract the pathophysiological mechanisms underpinning RA. For instance, in response to inflammatory stimuli, arachidonic acid is metabolized into pro-inflammatory lipids such as prostaglandin E2 (PGE2) and leukotriene B4 (LTB4). PGE2, synthesized by cyclooxygenase (COX)-1 and COX-2 enzymes in peripheral tissues including joints, promotes inflammation and pain. LTB4, meanwhile, acts as a potent chemoattractant, that recruits neutrophils to inflamed tissues (James et al. 2010). In RA, neutrophils are the predominant immune cells in the synovial fluid, significantly contributing to tissue damage and the perpetuation of the inflammatory process.

Despite the availability of various treatment options, many of these therapies are associated with limitations, including adverse effects (e.g., opportunistic infections, elevated liver enzymes, dyslipidemia, cytopenias, retinopathy, etc.); contraindications such as moderate/severe heart failure; and insufficient clinical benefits. Recent studies show that approximately 20–40% of patients fail to achieve significant improvement with these therapies (George et al. 2020). These challenges underscore the urgent need to develop more effective alternative therapies to improve the quality of life of patients with RA.

In recent years, the characterization and understanding of the gut microbiota have expanded, becoming a broad area of research, particularly in autoimmune diseases (Luckey et al. 2013). The gut microbiome plays a critical role in maintaining immune homeostasis, influencing both innate and adaptative immunity. Dysbiosis, an imbalance in the composition of gut microbial communities, has been implicated in various autoimmune diseases, including RA, inflammatory bowel disease, and multiple sclerosis (Dehner et al. 2019). Pathogenic microbes can compromise intestinal barrier integrity, increasing permeability and enabling the translocation of microbial antigens into the bloodstream. This process can trigger systemic inflammation and activate autoreactive immune cells, thereby contributing to the breakdown of immune tolerance. For example, microbiota can modulate host immune responses by activating antigen-presenting cells (APCs), such as dendritic cells (DCs), which in turn promote antigen presentation and cytokine production. These processes influence T cell differentiation and function (Xu et al. 2019). Furthermore, microbial metabolites such as short-chain fatty acids (SCFAs) play an essential role in immune regulation by modulating T regulatory cell (Treg) function and inhibiting pro-inflammatory cytokines like IL-6 and TNF-α. The influence of gut microbiota on the immune system goes beyond shaping the T cell repertoire and involves the excessive activation of plasma cells that produce antibodies, which plays a role in the development of autoimmunity (Willemze et al. 2019). Understanding the role of the gut microbiome in autoimmune diseases provides a valuable foundation for the development of microbiome-targeted interventions, including nutraceuticals, aiming at restoring immune balance and mitigating disease severity.

As previously mentioned, current RA treatments face significant challenges, including adverse effects, contraindications, and a lack of clinical response in a considerable percentage of patients. In this context, nutraceuticals emerge as a promising and potentially vital option to complement the management of RA. This work presents an updated review of the role of nutraceuticals in mitigating RA symptoms, aiming to provide an in-depth exploration of their mechanisms of action and their impact on the pathogenesis of the disease, with a focus on their modulatory functions in the inflammatory and immunological processes associated with RA.

### Current treatments for RA

The cornerstone of RA management has traditionally been the use of disease-modifying antirheumatic drugs (DMARDs), which aim to reduce inflammation, prevent joint damage, and improve functional outcomes. These therapies are categorized into conventional synthetic DMARDs (csDMARDs), biological DMARDs (bDMARDs), and targeted synthetic DMARDs (tsDMARDs). While csDMARDs remain the first-line treatment, bDMARDs have become essential for patients with moderate to severe RA or for those who do not respond adequately to csDMARDs (Sen et al. 2024; Findeisen et al. 2021; Ben Mrid et al. 2022).

#### Biological DMARDs in RA treatment

Biological DMARDs are a class of agents that target specific components of the immune system involved in the inflammatory cascade of RA. These include TNF-α inhibitors, IL-6 receptor antagonists, T-cell co-stimulation inhibitors, and B-cell depleting agents. TNF-α inhibitors such as adalimumab, etanercept, and infliximab—are among the most commonly used bDMARDs and have demonstrated efficacy in reducing disease activity and improving quality of life in patients with RA (Singh et al. 2009; Ben Mrid et al. 2022).

IL-6 receptor antagonists, like tocilizumab and sarilumab, have also shown effectiveness in RA treatment, particularly in patients who exhibit inadequate responses to TNF-α inhibitors. Tocilizumab has demonstrated superior efficacy compared to TNF inhibitors when used as monotherapy, without concurrent csDMARD, as shown in head-to-head trials with adalimumab (Findeisen et al. 2021; Ben Mrid et al. 2022).

**Fig. 2:**
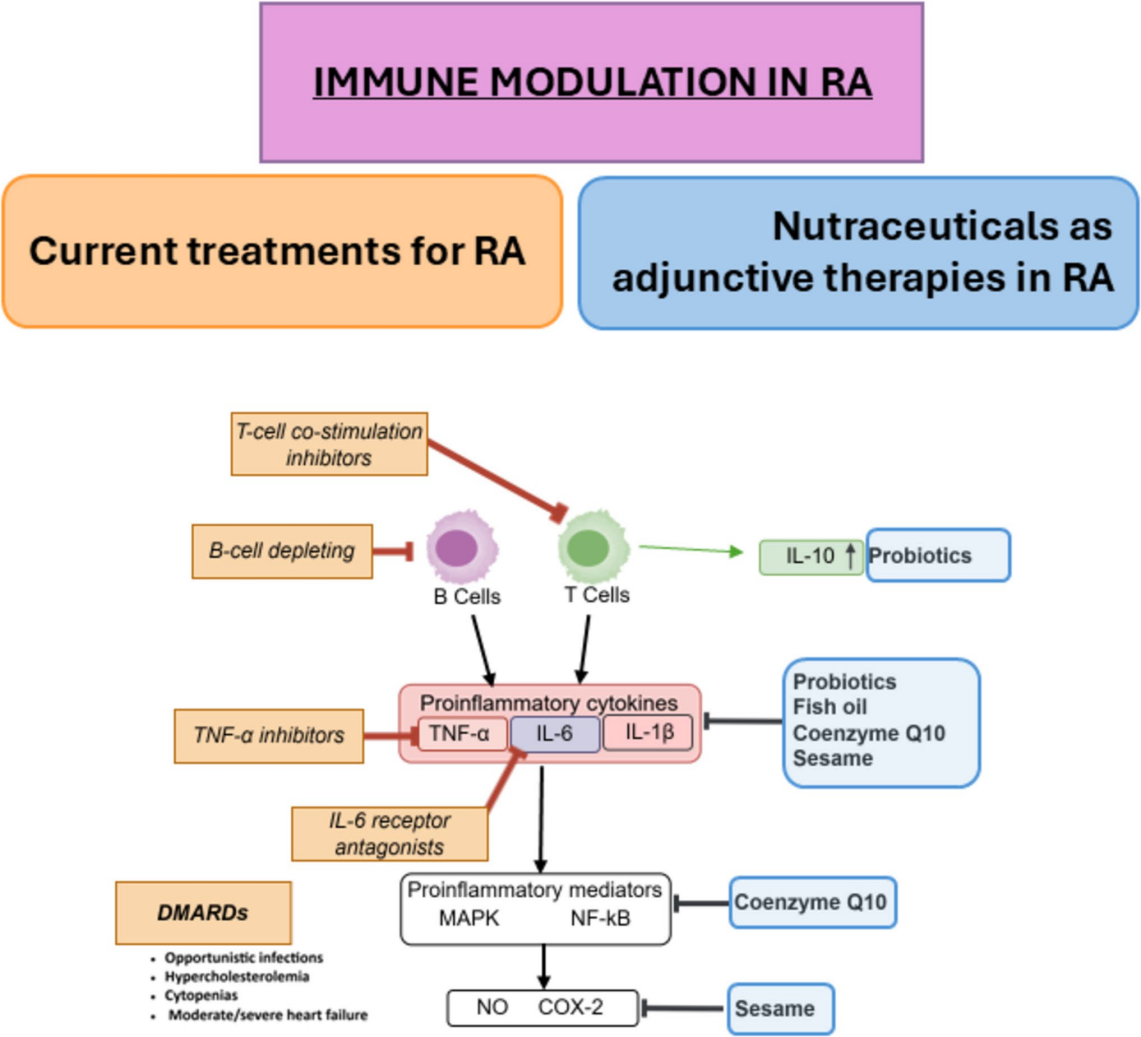
Diagram of the potential immune modulation by combining traditional treatments and the use of nutraceuticals as adjuvants. On the left, current RA therapies include T-cell co-stimulation inhibitors, B-cell depleting agents, TNF-α inhibitors, IL-6 receptor antagonists, and DMARDs, which are associated with adverse effects such as infections, cytopenias, and heart failure. On the right, nutraceuticals like probiotics, fish oil, coenzyme Q10, and sesame are shown to modulate immune responses, notably by enhancing anti-inflammatory cytokines like IL-10. Central pathways involve proinflammatory cytokines (TNF-α, IL-6, IL-1β) and signaling mediators (MAPK, NF-κB), which are targeted by both therapeutic approaches

T-cell co-stimulation inhibitors such as abatacept and B-cell depleting agents like rituximab offer alternative mechanisms of action for patients who do not respond to TNF-α inhibitors. Studies have shown that abatacept increases remission rates compared to methotrexate alone and to be effective in patients with disease refractory to TNF inhibitors (Findeisen et al. 2021; Ben Mrid et al. 2022) (Fig. 2).

### Limitations and challenges of biological DMARDs

Despite their efficacy, bDMARDs are associated with several limitations and challenges (Findeisen et al. 2021; Ben Mrid et al. 2022).

- *Adverse effects*: Common side effects include injection site reactions, headache, skin rashes, respiratory tract infections, and urinary tract infections. More serious adverse effects can include allergic reactions, hepatotoxicity, cancer, and serious infections such as tuberculosis, pneumonia, staphylococcal infections, and fungal infections (Ben Mrid et al. 2022).
- *Infection risk*: The use of bDMARDs increases the risk of serious infections, including reactivation of latent tuberculosis and opportunistic infections. The overall risk is further influenced by factors such as concomitant glucocorticoid use, advanced patient age, comorbidities, and the underlying increased risk associated with RA itself (Findeisen et al. 2021; Ben Mrid et al. 2022).
- *Discontinuation rates*: Discontinuation rates of bDMARDs vary, with some studies reporting high rates due to lack of efficacy or adverse effects. For instance, a retrospective observational study conducted in Australia reported a 51% discontinuation rate for TNF inhibitors over 7 years period (Findeisen et al. 2021).
- *Cost and accessibility*: The high cost bDMARDs remains a significant barrier to their widespread use, particularly in healthcare systems with limited resources. Although biosimilar drugs have been developed to mitigate costs, their availability varies across countries, and market integration challenges still persist (Findeisen et al. 2021).

### Need for alternative approaches

Given these limitations, there is an urgent need to explore alternative therapeutic strategies, such as the use of nutraceuticals. Nutraceuticals, including probiotics, omega-3 fatty acids, coenzyme Q10 (CoQ10), vitamin D, and polyphenols, have shown potential in modulating gut microbiota and inflammatory pathways. Exploring the interplay between these nutraceuticals, gut microbiota, and immune function in RA could offer novel strategies for disease management. This approach may complement existing therapies and provide additional options for patients, particularly those who exhibit inadequate responses to bDMARDs.

### Nutraceuticals: a novel treatment

The term “nutraceutical,” a portmanteau of the words “nutrition” and “pharmaceutical,” was coined in 1989 by Dr. Stephen DeFelice, a physician and scientist who founded the Foundation for Innovation in Medicine. According to DeFelice, a nutraceutical is defined as “a food (or part of a food) that provides medical or health benefits, including the prevention and/or treatment of disease (Kalra 2003; Alba et al. 2023).

Today, the term is widely used to describe products derived from food sources that offer health benefits beyond their basic nutritional value. These products, which contain active extracted from foods, are marketed with the intent to promote health and prevent or treat various diseases (Kalra 2003; Alba et al. 2023). Common examples include vitamins, minerals, plant-based compounds, amino acids, and other naturally occuring substances. The essence of the term lies in the convergence of nutritional benefits with the possibility that certain foods or supplements exhibit medicinal properties.

These properties position nutraceuticals as promising agents in the healthcare domain. In the context of RA, these compounds could act as effective adjuvants, helping to modulate the inflammatory response and reduce the oxidative damage associated with the disease. Moreover, as they are not linked to the severe side effects of conventional therapies, nutraceuticals represent an attractive alternative to overcome some of the current limitations of treatment, offering an additional pathway to improve clinical outcomes and quality of life.

Nutraceuticals are gaining a notable presence in the current market due to the wide range of benefits and options they provide for new treatments targeting autoimmune diseases such as RA. However, given their novel nature and developing research, a universally accepted definition and standardization framework for nutraceuticals remains lacking. This regulatory gap hinders the establishment of consistent guidelines for their clinical application. Nonetheless, their use continues to grow, particularly in countries such as the United States, Japan, and members of the European Union, where nutraceuticals have already achieved significant market presence (Hoti et al. 2022).

These compounds exert their biological effects through multiple molecular mechanisms, including modulation of the gut-immune axis, regulation of cytokine production, and direct influence on immune cell function. By targeting key inflammatory pathways, nutraceuticals offer a promising adjunctive therapeutic option, with the potential to reduce disease severity and improve patient outcomes.

### Rheumatoid arthritis and the gut microbiome

The gut microbiota has been recognized as a key environmental factor in the development of RA (Zhang et al. 2015). Recent research suggests that dysbiosis in RA is not just a secondary effect of chronic inflammation but may actively contribute to disease onset. Studies have demonstrated that RA patients exhibit significant alterations in their gut microbiota compared to healthy individuals. For instance, an increased abundance of Prevotella copri has been associated with early RA, potentially triggering autoimmune responses by activating antigen-presenting cells and enhancing the production of pro-inflammatory cytokines such as TFN-alpha and IL-6 (Pianta et al. 2017). Additionally, the expansion of Collinsella aerofaciens in RA patients has been linked to increased gut permeability through the downregulation of tight junction protein in epithelial cells and the induction expression of IL-17 network cytokines (Chen et al. 2016). This may facilitate the translocation of microbial antigens into the bloodstream, thereby amplifying systemic inflammation.

Animal models further support the role of the gut microbiome in RA pathogenesis. In the K/BxN mouse model, the absence of gut microbiota under germ-free (GF) conditions marked a reduction to autoimmune arthritis severity. This was associated with lower serum autoantibody levels, fewer splenic autoantibody-secreting cells, diminished germinal center formation, and a decrease in the splenic T helper (Th17) cell population (Wu et al. 2010). These findings underscore the complex interactions between gut microbes and the immune system, offering promising avenues for novel therapeutic strategies.

### Nutraceuticals in microbiome regulation and RA inflammation

The gut-immune axis represents a critical communication network between the intestinal microbiota and the host immune system, playing a central role in maintaining immune homeostasis. Disruptions in this axis have been linked to increased intestinal permeability, systemic inflammation, and the activation of autoreactive immune cells in RA. Nutraceuticals have been shown to positively influence the gut-immune axis by restoring microbial balance, strengthening gut barrier integrity, and modulating immune signaling pathways (Ban et al. 2022), (Fig. 2).

#### Probiotics

Probiotics are live microorganisms that, when administered in appropriate quantities, confer health benefits to the host (Alipour et al. 2014; Vaghef-Mehrabany et al. 2014). Primarily comprising bacteria, these microorganisms can modulate the gut microbiota and counteract dysbiosis (Derksen et al. 2017), a microbial imbalance that can contribute to local inflammatory phenomena, intestinal barrier dysfunction, and bacterial translocation into the bloodstream. Beyond improving digestive function, probiotics play a crucial role in strengthening the immune system by influencing both innate and adaptive immune responses (Alipour et al. 2014; Vaghef-Mehrabany et al. 2014).

Common probiotic strains used in human preparations include Lactobacillus, Bifidobacterium, Lactococcus, Streptococcus, and Enterococcus (Cannarella et al. 2021). These strains have been extensively studied for their safety and efficacy, positioning them as promising tools in managing microbiota-related disorders and inflammation.

Evidence from in vivo and in vitro studies has demonstrated that specific probiotic strains exert favorable immunomodulatory effects, suggesting their therapeutic potential in various pathological conditions. However, these effects largely depend on factors such as the strain used and the administered dose. Proposed mechanisms include modulation of the endogenous gut microbiota composition, reduction of oxidative and nitrosative stress, and regulation of systemic inflammation (Cannarella et al. 2021), (Fig. 2).

Furthermore, studies have shown that the anti-inflammatory effects of probiotics are evidenced by a significant reduction in proinflammatory cytokines, such as IL-1β, IL-2, IL-6, IL-12, and IL-17, interferon-gamma (IFN-γ), and TNF-α, along with an increase in regulatory cytokines such as IL-10 and transforming growth factor-beta (TGF-β) (Alipour et al. 2014; Vaghef-Mehrabany et al. 2014).

Since the gut microbiome plays a fundamental role in metabolic transformation, it is postulated that probiotics may influence the production of bacteriocins and SCFAs like butyric acid, which exert regulatory effects on inflammation and glucose transport (Zamani et al. 2016). These mechanisms positively affect the host’s physiological processes. However, studies have suggested that certain components of the bacterial cell wall could mimic human antigens (Derksen et al. 2017), potentially triggering immune responses directed toward the joints, thereby contributing to RA development or exacerbation. This highlights the complexity of the interaction between the microbiota and the immunological processes involved in RA pathogenesis.

#### Sesame

It has been observed that sesame, rich in compounds such as sesamol and sesamin, natural polyphenolic compounds, has been observed to exhibit antioxidant activity. This effect is attributed to its structure, as the hydroxyl group can donate hydrogen atoms to neutralize free radicals, thereby preventing oxidative cellular damage (Helli et al. 2019). Studies on neuroinflammation in mice have demonstrated that these compound’s anti-inflammatory and antioxidant properties are mediated through the regulation of signaling pathways involving AMP-activated protein kinase (AMPK), Sirtuin 1 (SIRT1), and NF-κB (Feng et al. 2022).

In vitro studies have shown that sesame seed extract reduces the number of monocyte-derived macrophages and inhibits low-density lipoprotein (LDL) oxidation (Deme et al. 2019). Studies have also shown a reduction in MMPs, the expression of TNF-α, and COX-2 (Helli et al. 2019). Additionally, sesamin has also been demonstrated to inhibit inflammation in intervertebral discs both in vitro and in vivo (Li and Lv, 2020), highlighting its potential in controlling inflammation, (Fig. 2).

#### Fish oil

Fish oil, abundant in omega-3 fatty acids such as alpha-linolenic acid (ALA), eicosapentaenoic acid (EPA), and docosahexaenoic acid (DHA), possesses anti-inflammatory properties by inhibiting inflammatory mediators and providing substrates that favor the synthesis of lipids, thereby reducing the inflammatory response.

However, EPA and DHA play a dual role in the regulation of inflammation. On one hand, they inhibit the production of inflammatory mediators by competing with arachidonic acid for COX enzymes, thereby reducing the synthesis of pro-inflammatory eicosanoids. (James et al. 2010). They also suppress peptidic inflammatory mediators such as TNF-α and IL-1β, aiding in controlling the initial phase of the inflammatory response (James et al. 2010; Proudman et al. 2015; Rajaei et al. 2015). Furthermore, the synthesis of oxylipins, bioactive lipids derived from EPA and DHA, reduces the production of inflammatory prostaglandins, thromboxanes, and leukotrienes, further enhancing their anti-inflammatory profile (Lindqvist et al. 2023).

Additionally, EPA and DHA serve as essential substrates for the biosynthesis of resolvins, a class of anti-inflammatory lipids that actively promote the resolution of inflammation (James et al. 2010; Proudman et al. 2015). This process begins when interactions between neutrophils, endothelial cells, and other cells at the inflammatory site, this interaction activates 15-lipoxygenase and 5-lipoxygenase enzymes, which catalyze the formation of resolvins (James et al. 2010). These molecules facilitate the resolution phase, a dynamic process that includes the cessation of neutrophil recruitment, phagocytosis of apoptotic cells by macrophages, and efficient clearance of apoptotic neutrophils from the inflammatory site, facilitating tissue recovery (Veselinovic et al. 2017).

Moreover, initial studies have demonstrated that fish oil supplementation can reduce the number of tender joints, decrease the use of NSAIDs, and potentially lower the risk of cardiovascular events in individuals with RA (James et al. 2010), (Fig. 2).

#### Coenzyme Q10

CoQ10, classified as a fat-soluble vitamin, is intrinsically present in the diet and is endogenously synthesized by all cells in the body (Mugoni et al. 2013). As an antioxidant, CoQ10 plays a crucial role in redox processes within cell membranes, neutralizing free radicals and regenerating tocopherol (Kagan et al. 2000; Ouchi et al. 2010), complemented by its anti-inflammatory properties. Studies have shown that CoQ10 suppresses TNF-α gene expression in animal models, likely mediated through NF-kB dependent mechanisms, thereby highlighting its anti-inflammatory properties (Schmelzer et al. 2008). Similarly, research has demonstrated a notable reduction in TNF-α levels following CoQ10 supplementation, although no notable changes were observed in IL-6 levels (Abdollahzad et al. 2015).

Additionally, CoQ10 fulfills essential roles as an electron carrier, a protein translocator, and a contributor to the biosynthesis of vitamin E. These roles underscore its importance as a key modulator of oxidative stress, Fig. 2. Studies have shown that cellular deficiency of CoQ10 can lead to an increase in mitochondrial density, triggering the development of myopathies and neuropathies (Wang and Hekimi, 2022).

#### Vitamin D

Vitamin D, a fat-soluble vitamin, possesses a molecular structure analogous to that of steroids and exhibits endocrine activity, thereby allowing it to be classified as a hormone (Rolando and Barabino, 2023). Although its primary function is related to the regulation of calcium and phosphorus metabolism, crucial for bone mineralization (Rolando and Barabino, 2023; Herly et al. 2018), its function extends well beyond skeletal health.

Furthermore, it also plays a significant role as an immunomodulator. Studies have shown that insufficient levels of vitamin D may promote a Th1-and Th17-mediated autoimmune response, characterized by elevated production of proinflammatory cytokines such as IFN-γ, IL-1, IL-6, and IL-17 (Herly et al. 2018). Additionally, T lymphocytes, DCs, monocytes, and macrophages express both vitamin D receptors and the enzymes necessary for its activation. The expression of these receptors and enzymes varies according to the activation state of the cells (Herly et al. 2018).

In addition, vitamin D regulates biological processes such as autophagy and oxidative stress pathways (Weiss and Litonjua, 2017), emphasizing its role in modulating inflammatory responses, controlling antigen-presenting cells, and regulating immune tolerance, thereby preventing the promotion of chronic inflammation or the development of an autoimmune response.

## Methods

### Study design

An exhaustive review of the most relevant and up-to-date scientific literature was conducted, in accordance with the guidelines of the *Preferred Reporting Items for Systematic Reviews and Meta-Analyses* (PRISMA) guidelines (Page et al. 2021), which were used as a framework to structure the present review.

### Selection criteria

#### Inclusion criteria

- Randomized controlled trials (RCTs).
- Studies involving patients diagnosed with rheumatoid arthritis (RA).
- Studies investigating treatments utilizing the following nutraceuticals: probiotics, sesame, fish oil, CoQ10, or Vitamin D.
- Studies published from 2014 onward.
- Studies conducted in human populations.

#### Exclusion criteria

- Studies currently recruiting patients or ongoing studies.
- Studies addressing autoimmune diseases other than RA.
- Studies lacking a therapeutic approach involving the use of nutraceuticals.
- Studies where the investigated nutraceuticals differ from probiotics, sesame, fish oil, CoQ10, or Vitamin D.
- Studies conducted on pediatric populations.

### Study types

This study includes randomized controlled trials (RCTs) published from 2014 to May 1, 2025. Trials investigating the effectiveness of specific nutraceuticals in the treatment of RA, in comparison to conventional treatments, were selected.

### Participants

The participants included were adult males and females diagnosed with RA, according to the 2010 American College of Rheumatology (ACR) criteria. Most participants had been living with the disease for at least six months and exhibited disease activity levels ranging from low to severe, as assessed by the Disease Activity Score-28 (DAS28). Participants were undergoing antirheumatic treatment without the use of NSAIDs or cytokine inhibitors and maintained the same medication regimen for at least 3 months prior to the initiation of supplementation. Studies were excluded if patients had severe concomitant gastrointestinal or metabolic diseases, were pregnant or breastfeeding, or had hepatorenal conditions, among other exclusions.

### Interventions

Patients in the selected studies were subjected to various interventions alongside their usual antirheumatic therapy, including:

- Supplementation with probiotics (Lactobacillus acidophilus, Bifidobacterium bifidum, and/or Lactobacillus casei).
- Daily sesame intake (200 mg/day).
- Consumption of Omega-3 Cardio (300 mg DHA, 200 mg EPA, and 100 mg other n-3 PUFAs).
- Use of Primrose Oil (1300 mg evening primrose oil, 949 mg linoleic acid, and 117 mg gamma-linolenic acid).
- CoQ10 supplementation (100 mg/day).
- Oral administration of Vitamin D at doses of 100,000 IU and 300,000 IU.

### Outcome variables

- *DAS28*: The DAS28 score assesses disease activity in RA patients by measuring inflammatory blood markers, such as erythrocyte sedimentation rate (ESR) or C-reactive protein (CRP) and incorporates the patient’s subjective perspective of their overall well-being, measured by a Visual Analog Scale (VAS) (Pisaniello et al. 2022).
- *HAQ (Health Assessment Questionnaire)*: The HAQ is used to assess functional disability and quality of life in affected individuals (Health Assessment Questionnaire, 1996).
- *US-7 (Ultrasound-7)*: This scale evaluates disease activity in RA, based on ultrasound assessment of seven joints in each hand and wrist (Ohrndorf et al. 2013).
- *Cytokines*: Cytokines play a critical role in regulating immune responses and the inflammatory cascade. Pro-inflammatory cytokines include TNF-α, IL-1, IL-12, and IL-6, while regulatory cytokines like IL-10 or anti-inflammatory cytokines (Th2 pattern) such as IL-4 and IL-5 counterbalance pro-inflammatory effects (George et al. 2020).
- *MMPs*: Elevated levels of specific MMPs, including MMP-1, MMP-3, and MMP-13, have been identified as markers associated with disease progression and joint degradation (Burrage et al. 2006).
- *COX-2*: Quantitative evaluation of COX-2 is a relevant marker for assessing inflammatory activity and joint involvement (James et al. 2010).
- *Homeostasis Model Assessment of Insulin Resistance (HOMA-IR) and Homeostasis Model Assessment of Beta-Cell Function (HOMA-B)*: These indices assess insulin resistance and beta-cell function, respectively. Their inclusion is pertinent due to the increased risk of type 2 diabetes among RA patients (Zamani et al. 2016).
- *Total antioxidant capacity (TAC)*: TAC measures the body’s ability to neutralize free radicals and other oxidative compounds. Oxidative stress contributes to chronic inflammation, cartilage and bone degradation, and synovial proliferation (Kagan et al. 2000). Additionally, malondialdehyde (MDA), a product of lipid peroxidation, serves as an indirect measure of oxidative stress (Abdollahzad et al. 2015).

### Search strategies

For this review, a comprehensive search was conducted across several scientific databases, including PubMed and Web of Science (WOS), up to May 1, 2025. Selection criteria were stringent, limiting documents to those published within the last 10 years, and to RCTs in PubMed or, alternatively, “clinical trials” in WOS, as it was not possible to filter by RCTs in the latter. The aim was to derive conclusions supported by the most current scientific literature. Specific keywords were used for the search, such as: “Rheumatoid arthritis AND probiotics,” “Rheumatoid arthritis AND sesamin,” “Rheumatoid arthritis AND fish oil,” “Rheumatoid arthritis AND CoQ10,” and “Rheumatoid arthritis AND Vitamin D.” These terms were combined using the boolean operator “AND”, Table 1.

**Table 1:** Representative scheme of the bibliographic search strategy used

Databases	MESH Terms and Boolean Operators	Results
PubMed	Rheumatoid arthritis AND probiotics	8
	Rheumatoid arthritis AND sesamin	2
	Rheumatoid arthritis AND “fish oil”	6
	Rheumatoid arthritis AND CoQ10	2
	Rheumatoid arthritis AND “Vitamin D”	12
WOS	Rheumatoid arthritis AND probiotics	14
	Rheumatoid arthritis AND sesamin	2
	Rheumatoid arthritis AND “fish oil”	21
	Rheumatoid arthritis AND CoQ10	2
	Rheumatoid arthritis AND “Vitamin D” 2	29

MESH: Medical Subject Headings. WOS: Web of Science

### Classification of study quality

The methodological quality of each clinical trial included in this review was assessed using the PEDro scale. This scale consists of 11 items, with scores varying depending on whether each item is fulfilled. Regarding the risk of bias and the quantification of study quality, a score ranging from 6 to 8 was considered indicative of moderate methodological quality with a moderate risk of bias, while a score between 9 and 11 indicated high methodological quality with a low risk of bias.

**Table 2:** Summary of nutraceuticals as therapies for RA

Nutraceutical	Study	Sample size	Mechanism of action	Effects on RA	Limitations
Probiotics	Alipour et al. (2014)	N = 46, EG = 22, CG = 24	Modulation of gut microbiota, ↑SCFAs downgrade levels of pro inflammatory cytokines ↓NF-κB/NLRP3.Enhances Treg differentiation regulates ↑IL-10, ↓IL-12/TNF-α	↓DAS28 (*p* = 0.002). ↓hs-CRP (*p* < 0.05). ↓Swollen joints (*p* < 0.05)	Short duration (8 weeks). Effects not generalizable to other probiotics
	Vaghef-Mehrabany et al. (2014)	N = 46, EG = 22, CG = 24	Normalizes gut microbiota. ↓proinflammatory cytokines (TNF-α, IL-6, IL-12) interaction with Toll-like receptors on dendritic cells, promoting regulatory T cells (Tregs)	↓ DAS28 (*p* < 0.01). ↓ TNF-α (*p* < 0.01). ↓ IL-6 (*p* < 0.05). ↓ IL-12 (*p* < 0.01). ↑ IL-10 ( *p* < 0.05). ↑ IL-10/IL-12 (*p* = 0.01)	Lack of fecal recovery data to confirm colonization
	Zamani et al. (2016)	N = 60, EG = 30, CG = 30	Anti-inflammatory effects production of bacteriocins and short-chain fatty acids downregulation of systemic inflammation modulation of gut microbiota and immune responses	Significant decrease in; ↓ DAS-28 (*p* = 0.01), ↓ insulin levels (*p* = 0.03), ↓ HOMA-B (*p* = 0.03), ↓ CRP (*p* < 0.001), ↓ LDL-cholesterol (*p* = 0.07)	Short duration (8 weeks). No measurement of inflammatory cytokines (TNF-α, IL-6)
	Zamani et al. (2017)	N = 54, EG = 27, CG = 27	Modulates gut microbiota. Reduces proinflammatory cytokines (TNF-α, IL-6). Production of short-chain fatty acids (SCFA) enhanced antioxidant activity (↑ glutathione) improved insulin sensitivity	↓ Decrease in: hs-CRP (*p* = 0.001), DAS-28 (*p* < 0.001), VAS pain (*p* < 0.001), insulin (*p* = 0.01), HOMA-IR (*p* = 0.03), HOMA-B (*p* = 0.01). ↑ Increase in plasma NO (*p* = 0.008) and plasma GSH (*p* = 0.005)	Duration (8 weeks). Lack of fecal microbiota analysis. No measurement of inflammatory cytokines (TNF-α, IL-6)
Sesame	Helli et al. (2019)	N = 44, EG = 22, CG = 22	Reduced inflammation and cartilage degradation (Inhibits NF-κB pathway, reduces COX-2 activity.) enhanced antioxidant defenses (↓ Reduces oxidative stress)	Significant decrease in MMPs (*p* = 0.039), hyaluronidases (p = 0.045), CRP (*p* = 0.046), TNF-α (*p* = 0.039), and COX-2 (*p* = 0.004)	No significant changes in the levels of IL-6 and IL-1β
Fish oilSoubrier	Veselinovic et al. (2017)	N = 60, EG 1 = 20, EG 2 = 20, CG = 20	Inhibition of inflammatory mediators (↓ CRP, ↓ ESR), synergistic effect of n-3 PUFA + GLA on lipid profiles	Significant decrease in ↓ DAS-28 in fish oil group (*p* < 0.001), in groups I and II was a decrease in ↓ Tender joints (*p* < 0.001), ↓ VAS pain (*p* < 0.001), ↓ CRP (*p* ≤ 0.001)	DAS-28 is only significantly decreased in fish oil group no placebo control
	Dawczynski et al. (2018)	N = 38, EG = 19, CG = 19	Modulates lipid mediator balance, synthesis of anti-inflammatory DHA-derived mediators	There was a significant decrease in ↓US-7 scale, ↓ DAS28 (*p* = 0.072)	No significant improvement was observed in the following parameters: CRP, ESR, HAQ
Coenzyme Q10	Nachvak et al. (2019),	N = 54, EG = 27, CG = 27	Suppression of TNF-α mediated by NF-kB1. Antioxidant properties mitigate inflammation	A significant reduction in TNF-α and MDA levels	Nno significant changes in IL-6 and CAT
	Abdollahzad et al. (2015)	N = 44, EG = 22, GC = 22	The results indicated a significant difference in MMP-3. A significant reduction in TNF-α levels was observed. No significant changes were observed in IL-6	Statistically significant improvements in disease activity (DAS-28) (*p* < 0.001), joint counts, pain, and MMP-3 levels (*p* = 0.027)	Non-significant changes in some inflammatory markers
Vitamin D	Soubrier et al. (2018)	N = 59, EG = 29, GC = 30	Modulates immune response via vitamin D receptor (VDR), reduces inflammation, and improves calcium metabolism	The results revealed a significant improvement in HAQ (*p* = 0.046) along with a decrease in ESR (*p* = 0.002) and CRP (*p* = 0.04)	The improvement in DAS28-ESR was not statistically significant
	Buondonno et al. (2017)	N = 36, EG = 18, CG = 18	Regulation of inmune response (IL-23, IL-6) and inhibition of Th17 differentiation. Oxidative stress and modulation of autophagy	A significant increase was observed of 25-OH (*p* = 0.015), TNF-α, IL-23, IL-6	The changes in DAS-28 and PCR are not statistically significant. Baseline Vitamin D levels are not stratified

## Results

**Fig. 3:**
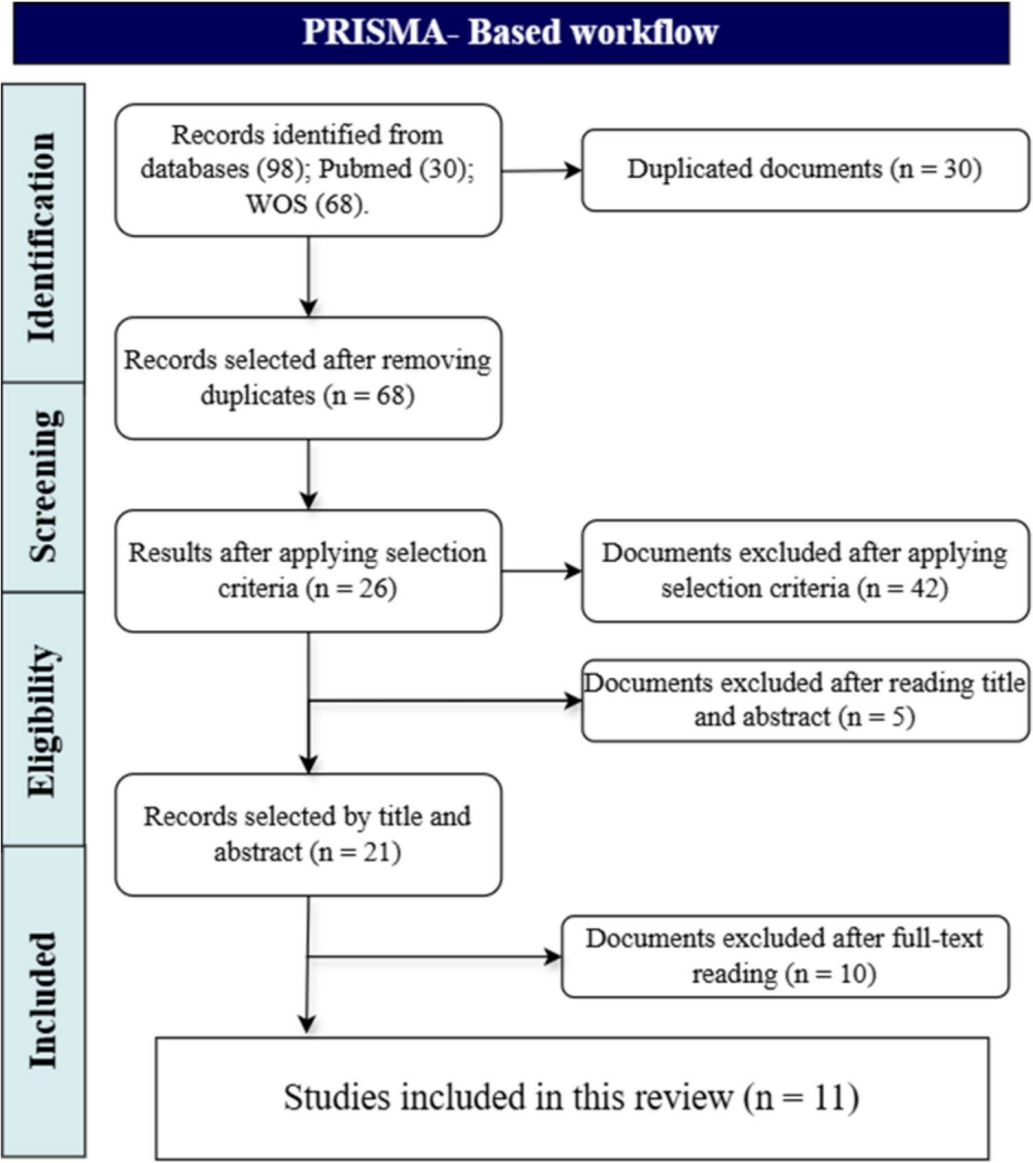
Flowchart of the identified studies. This PRISMA-based flow diagram outlines the systematic review process. It begins with the identification of 98 records from PubMed and Web of Science (WOS), followed by the removal of 30 duplicates. After screening 68 records, 42 were excluded based on selection criteria, leaving 26 for eligibility assessment. Of these, 5 were excluded after title and abstract review, and 10 more after full-text evaluation. Ultimately, 11 studies were included in the final review

After completing the initial search phase, a total of 98 records were identified. Thirty duplicates were subsequently removed, reducing the total to 68 records. Following the application of inclusion criteria, 26 studies met the established requirements. After a thorough review of titles and abstracts, this number was narrowed down to 21. Finally, after detailed analysis of the full texts, 11 studies were selected as relevant for the purpose of this research. The flow of study identification, in accordance with PRISMA guidelines, is depicted in Fig. 3.

### Studies included

This review includes 11 randomized controlled trials (RCTs) comprising a total of 541 participants from various geographical regions. Seven studies were conducted in Iran (Alipour et al. 2014; Vaghef-Mehrabany et al. 2014; Zamani et al. 2016, 2017; Helli et al. 2019; Abdollahzad et al. 2015; Nachvak et al. 2019), totaling 348 participants. The remaining studies were conducted in Serbia (Veselinovic et al. 2017) (60 participants), Germany (Dawczynski et al. 2018) (38 participants), Italy (Buondonno et al. 2017) (36 participants), and France (Soubrier et al. 2018) (59 participants). Table 2 provides a detailed summary of the specific characteristics of each study, including relevant information on the study population, the interventions performed in the different participant groups, the variables analyzed, and the main outcomes reported.

### Participant data

The largest studies included 60 participants, as seen in studies (Zamani et al. 2016; Veselinovic et al. 2017), while the smallest study involved 36 participants (Buondonno et al. 2017). In all cases, female participants predominated, with five studies exclusively involving women. The demographic data revealed an average age of approximately 52 years in studies reporting this information. However, variability was observed, with Veselinovic et al. reporting the highest mean age of 63.1 ± 9.6 years (Veselinovic et al. 2017), and the youngest participants having a mean age of 42.78 years (Alipour et al. 2014; Vaghef-Mehrabany et al. 2014).

### Interventions

All studies examined two comparison arms, except for one with three arms (Veselinovic et al. 2017). All trials compared the addition of a specific nutraceutical to conventional RA therapy. Four studies evaluated the effectiveness of specific probiotics (Lactobacillus acidophilus, Bifidobacterium bifidum, and/or Lactobacillus casei) combined with standard treatment versus conventional therapy alone (Alipour et al. 2014; Vaghef-Mehrabany et al. 2014; Zamani et al. 2016, 2017). Additionally, one study investigated sesame supplementation combined with standard treatment, as compared to standard treatment alone (Helli et al. 2019). Two other studies employed fish oils, such as omega-3, evening primrose oil, or microalgae oil, alongside baseline treatment (Veselinovic et al. 2017; Dawczynski et al. 2018). Two further studies examined CoQ10 supplementation in addition to conventional therapy (Abdollahzad et al. 2015; Nachvak et al. 2019). Lastly, two studies evaluated vitamin D supplementation combined with conventional treatment compared to conventional therapy alone (Buondonno et al. 2017; Soubrier et al. 2018).

Regarding intervention duration, six RCTs lasted 8 weeks (Alipour et al. 2014; Vaghef-Mehrabany et al. 2014; Zamani et al. 2016, 2017; Helli et al. 2019; Abdollahzad et al. 2015; Nachvak et al. 2019), two studies extended over 3 months (Veselinovic et al. 2017; Buondonno et al. 2017), and the remaining studies varied: one lasted 6 weeks (Helli et al. 2019), another 6 months (Soubrier et al. 2018), and the final RCT 30 weeks (Dawczynski et al. 2018).

### Outcome measures

The most frequently studied variables included the DAS-28 scale in nine studies (Alipour et al. 2014; Vaghef-Mehrabany et al. 2014; Zamani et al. 2016, 2017; Veselinovic et al. 2017; Nachvak et al. 2019; Dawczynski et al. 2018; Buondonno et al. 2017; Soubrier et al. 2018), C-reactive protein (CRP) and/or erythrocyte sedimentation rate (ESR) in eight studies (Alipour et al. 2014; Zamani et al. 2016, 2017; Helli et al. 2019; Veselinovic et al. 2017; Dawczynski et al. 2018; Buondonno et al. 2017; Soubrier et al. 2018), and various cytokines in five trials (Alipour et al. 2014; Vaghef-Mehrabany et al. 2014; Helli et al. 2019; Abdollahzad et al. 2015; Dawczynski et al. 2018). HOMA-IR and HOMA-B were evaluated in two trials (Zamani et al. 2016, 2017), the Health Assessment Questionnaire (HAQ) in two studies (Dawczynski et al. 2018; Soubrier et al. 2018), and matrix metalloproteinases (MMPs) in two studies (Helli et al. 2019; Nachvak et al. 2019). The total antioxidant capacity (CAT) was assessed in three trials (Zamani et al. 2016, 2017; Abdollahzad et al. 2015), while the US-7 questionnaire was evaluated in one study (Dawczynski et al. 2018). COX-2 was assessed in one trial (Helli et al. 2019), and malondialdehyde (MDA) in another (Abdollahzad et al. 2015).

### Follow-up

All studies performed outcome measurements at the beginning and end of the treatment period. However, none conducted post-intervention follow-up after treatment completion. Regarding in-treatment follow-up, specifically for probiotic interventions, no intermediate measurements were recorded between the start and end of the 8 weeks treatment periods (Alipour et al. 2014; Vaghef-Mehrabany et al. 2014; Zamani et al. 2016, 2017). In the trial evaluating sesame, weekly phone follow-ups were conducted to monitor adherence (Helli et al. 2019) but no analytical follow-up occurred until 6 weeks after the study commenced, when the trial was considered complete. For the two fish oil trials, one lacked intermediate follow-up between the start and end of the study, which lasted 3 months (Veselinovic et al. 2017). In contrast, the other trial had meticulous follow-up, including daily recording of pain, dietary intake, and medication use, as well as blood analyses to assess disease activity at weeks 10 and 20, during a total follow-up of 30 weeks (Dawczynski et al. 2018). In studies involving CoQ10, no additional follow-up was performed between the start and end of treatment (Abdollahzad et al. 2015; Nachvak et al. 2019). Regarding vitamin D studies, one had no follow-up after the study period, limiting it to the initial and final follow-up at 3 months (Buondonno et al. 2017). However, in the other study, vitamin D levels were monitored before the trial began, with doses adapted according to each participant’s deficiency level, and participants received fixed doses of vitamin D for the following 6 months (Soubrier et al. 2018).

### Methodological quality according to the PEDro scale

Of the eleven RCTs included, two exhibited moderate methodological quality (Abdollahzad et al. 2015; Dawczynski et al. 2018), with scores of 7 and 8, respectively. The remaining nine trials demonstrated high methodological quality: three studies received a score of 9 (Alipour et al. 2014; Vaghef-Mehrabany et al. 2014; Nachvak et al. 2019), five achieved a score of 10 (Zamani et al. 2016, 2017; Veselinovic et al. 2017; Buondonno et al. 2017; Soubrier et al. 2018), and one obtained a perfect score of 11 (Helli et al. 2019). Therefore, no studies displayed low methodological quality according to the PEDro scale. All studies fulfilled the criteria for subject selection, randomization, concealed allocation, initial homogeneity of groups, participant blinding, between-group comparisons, and accurate outcome measures with variability. However, one study did not apply evaluator blinding (Abdollahzad et al. 2015), and five studies did not employ therapist blinding (Zamani et al. 2016, 2017; Veselinovic et al. 2017; Abdollahzad et al. 2015; Dawczynski et al. 2018). Additionally, five studies did not meet the 85% threshold for participant completion (Alipour et al. 2014; Vaghef-Mehrabany et al. 2014; Abdollahzad et al. 2015; Nachvak et al. 2019; Dawczynski et al. 2018) and seven studies did not conduct intention-to-treat analysis (Alipour et al. 2014; Vaghef-Mehrabany et al. 2014; Abdollahzad et al. 2015; Nachvak et al. 2019; Dawczynski et al. 2018; Buondonno et al. 2017; Soubrier et al. 2018).

### Effect of interventions

The results from the probiotic intervention groups demonstrated notable consistency across the four trials, with all studies reporting a general decrease in the DAS-28 (Alipour et al. 2014; Vaghef-Mehrabany et al. 2014; Zamani et al. 2016, 2017). Regarding CRP, a significant reduction was observed in the three studies that evaluated this parameter (Alipour et al. 2014; Zamani et al. 2016, 2017). As for cytokine profiles, including TNF-α, IL-6, IL-10, IL-12, and IL-1β, a significant reduction in TNF-α levels was observed in both studies in which it was measured (Alipour et al. 2014; Vaghef-Mehrabany et al. 2014). In the case of IL-6, one study reported a significant reduction, while the other found no significant change (Alipour et al. 2014). IL-1β did not show significant changes in either study10,11. Regarding IL-10 and IL-12, a significant increase and decrease, respectively, were recorded in the aforementioned trials (Alipour et al. 2014; Vaghef-Mehrabany et al. 2014). The HOMA-B, HOMA-IR, and CAT parameters were evaluated in two of the four RCTs that investigated probiotics (Zamani et al. 2016, 2017). Consistent results were found for HOMA-B, with a significant decrease in both studies (Zamani et al. 2016, 2017). In contrast, the CAT response was divergent, but stable in both articles, with no significant differences recorded (Zamani et al. 2016, 2017). On the other hand, HOMA-IR showed a significant decrease in one study (Zamani et al. 2017), whereas the other found no statistically significant difference (Zamani et al. 2016).

The only trial included in this review that investigated the effects of sesame supplementation reported statistically significant results in the reduction of various parameters, including MMPs, hyaluronidases, CRP, TNF-α, and COX-2 (Helli et al. 2019). However, no significant changes were noted in IL-6 and IL-1β levels.

Regarding the results of the studies investigating fish oil supplementation, findings were heterogeneous (Veselinovic et al. 2017; Dawczynski et al. 2018). One RCT showed a significant reduction in DAS-28 in the experimental groups (Veselinovic et al. 2017), while the other showed no significant improvement (Dawczynski et al. 2018). Similarly, significant reductions in ESR and CRP were found in one study (Veselinovic et al. 2017), while no improvements were observed in the other (Dawczynski et al. 2018). Finally, a significant decrease in the US-7 score was observed in one of the studies (Dawczynski et al. 2018).

Among the studies evaluating CoQ10, one reported a significant decrease in DAS-28 (Nachvak et al. 2019). For MMPs, a significant reduction in MMP-3 was recorded, but not in MMP-1 (Nachvak et al. 2019). Another study examining cytokine levels observed a significant decrease in TNF-α, but not significant change in IL-6 levels (Abdollahzad et al. 2015).

Finally, regarding parameters related to oxidative stress, such as MDA and CAT, evaluated in one of the two RCTs. A significant reduction in MDA levels was identified, while no significant changes were observed in CAT (Abdollahzad et al. 2015).

Lastly, with regard to vitamin D supplementation, the following findings were noted (Buondonno et al. 2017; Soubrier et al. 2018). Both RCTs did not show a statistically significant difference in DAS-28 reduction (Buondonno et al. 2017; Soubrier et al. 2018). Regarding CRP, a significant decrease was observed in one of the trials (Soubrier et al. 2018), while no changes were observed in the other (Buondonno et al. 2017). In one of the studies, various cytokines (TNF-α, IL-23, and IL-6) were assessed, all of which showed a significant increase (Buondonno et al. 2017).

In conclusion, the HAQ score showed a significant improvement in one of the included studies (Soubrier et al. 2018).

### Pharmacokinetics and safety

The clinical effects of nutraceuticals in RA depend not only on their anti-inflammatory and antioxidant properties but also on their pharmacokinetic characteristics and safety profiles. Importantly, in the 11 randomized controlled trials included in this review, comprising a total of 541 participants, the interventions were administered at standardized doses and were consistently well tolerated, with no reports of severe adverse events (Alipour et al. 2014; Vaghef-Mehrabany et al. 2014; Zamani et al. 2016, 2017; Helli et al. 2019; Abdollahzad et al. 2015; Nachvak et al. 2019; Veselinovic et al. 2017; Dawczynski et al. 2018; Buondonno et al. 2017; Soubrier et al. 2018).

#### Probiotics

Supplementation with *Lactobacillus acidophilus, Bifidobacterium bifidum,* and/or *Lactobacillus casei* (Alipour et al. 2014; Vaghef-Mehrabany et al. 2014; Zamani et al. 2016, 2017; Nachvak et al. 2019) does not conform to traditional pharmacokinetic patterns, as probiotic strains exert their effects locally within the gut. Their clinical activity depends on transient colonization and modulation of gut microbiota composition rather than systemic absorption. Across RCTs, daily supplementation was well tolerated, with only mild gastrointestinal symptoms reported.

#### Sesame

In the RCT conducted by Helli et al. (2019), patients received 200 mg/day of sesame supplementation (Helli et al. 2019). Sesame lignans (sesamin, sesamol) are lipid-soluble and undergo hepatic metabolism into conjugated enterolignans (Shi et al. 2022). Although human data indicate limited bioavailability, measurable plasma metabolites confirm systemic exposure (Khalesi et al. 2016; Mostashari and Mousavi Khaneghah, 2024). Clinically, sesame was well tolerated, with no adverse effects beyond occasional gastrointestinal discomfort, supporting its favorable safety profile (Helli et al. 2019; Khalesi et al. 2016).

#### Fish oil and primrose oil

In the study by Veselinovic et al. (2017), patients consumed omega-3 Cardio gel capsules containing 300 mg DHA, 200 mg EPA, and 100 mg other n-3 PUFAs daily (Veselinovic et al. 2017). In the trial by Soubrier et al. (2018), patients received 1300 mg/day of evening primrose oil (949 mg linoleic acid, 117 mg gamma-linolenic acid) (Soubrier et al. 2018). Both interventions are absorbed in the small intestine and incorporated into plasma lipids and cell membranes, where they exert sustained immunomodulatory and anti-inflammatory effects (Bodur et al. 2025; Farag et al. 2023). Across RCTs, these supplements were well tolerated, with only mild gastrointestinal symptoms reported. Notably, no increased bleeding risk was observed at the studied doses.

#### Coenzyme Q10

In the study by Abdollahzad et al. (2015), patients received 100 mg/day of CoQ10 (Abdollahzad et al. 2015). As a lipophilic compound, CoQ10 requires bile salts for intestinal absorption, and its oral bioavailability is limited, though improved by lipid carriers. It is distributed to metabolically active tissues and metabolized in the liver (Mantle and Dybring 2020). No adverse effects were reported at this dose; in other contexts, mild gastrointestinal symptoms remain the most common side effect.

#### Vitamin D

In the trial by Buondonno et al. (2017), patients received high-dose oral vitamin D at 100,000 IU and 300,000 IU (Buondonno et al. 2017). Vitamin D is absorbed with dietary fat, hydroxylated in the liver to 25(OH)D, and subsequently activated in the kidney to 1,25(OH)2D (Giustina et al. 2024; Deepika et al. 2025). With a half-life of 2–3 weeks, supplementation at these doses achieved stable serum concentrations (Giustina et al. 2024). No clinically relevant adverse effects were observed in RA patients, although chronic overdosing is associated with hypercalcemia and nephrolithiasis (Buondonno et al. 2017).

In summary, across the 11 RCTs included (541 participants), nutraceutical supplementation was safe and well tolerated, with only occasional minor gastrointestinal events reported. No serious adverse events were observed. However, pharmacokinetic limitations, particularly the poor bioavailability of lignans and CoQ10, remain a challenge to achieving consistent clinical efficacy. Future trials should incorporate pharmacokinetic endpoints and standardized safety assessments to optimize dosage, formulation, and long-term safety in RA management.

## Discussion

The objective of this review was to analyze the effectiveness of nutraceuticals in alleviating symptoms in patients with RA. The results obtained from the review reveal a variety of significant findings that highlight the therapeutic potential of different interventions in RA management. This critical analysis focuses on the efficacy of several agents, including probiotics, sesame, fish oil, CoQ10, and vitamin D, in modulating clinical and biological markers associated with RA. Probiotics emerge as a promising intervention in the treatment of RA, with consistent reductions in DAS-28 and CRP levels observed across the reviewed RCTs (Alipour et al. 2014; Vaghef-Mehrabany et al. 2014; Zamani et al. 2016, 2017). The decrease in TNF-α along with an increase in IL-10 in response to probiotic therapy, suggests possible anti-inflammatory and regulatory mechanisms, highlighting the potential of probiotics to modulate systemic inflammation and reduce disease activity in RA. However, it is crucial to consider the variability in the effects on other cytokines such as IL-6, with one study reporting a significant decrease (Vaghef-Mehrabany et al. 2014), and another showing no significant change (Alipour et al. 2014). This inconsistency underscores the need for further research to fully understand the mechanisms of cytokines in relation to RA. Furthermore, large variability in HOMA-B and HOMA-IR results has been observed between studies, which could be attributed to differences in the composition of the probiotics used (Zamani et al. 2016, 2017). Notably, one study included as a component of the daily probiotic (Zamani et al. 2017), which may have influenced the outcomes and the characteristics of the participants.

Regarding CAT, which, as mentioned above, evaluates the ability to neutralize free radicals and other oxidative compounds that, in turn, promote chronic inflammation, cartilage degradation, and other pathological processes, the studies in which it was analyzed described a lack of significant reduction in this parameter following probiotic intervention. This raises questions about the efficacy of such interventions in mitigating oxidative stress in RA (Zamani et al. 2016, 2017). Despite the demonstrated importance of CAT in the body’s antioxidant defense system, its lack of significant response suggests the need for additional research to better understand the mechanisms by which probiotics may modulate oxidative stress in RA. It is important to consider that the antioxidant response is a complex process involving multiple enzymes and antioxidant systems. Therefore, the lack of significant changes in CAT activity does not completely rule out the potential role of probiotics in modulating oxidative stress in RA. Additional research using other markers of oxidative stress may be necessary to fully understand the effects of probiotics on the antioxidant response in RA. Furthermore, the lack of a significant reduction in CAT activity highlights the need to consider complementary therapeutic approaches that can more effectively address oxidative stress in RA. This could involve combining specific antioxidant interventions with probiotics or other therapies to optimize oxidative stress control and improve clinical outcomes in RA patients.

On the other hand, sesame, although investigated in a single study, demonstrated beneficial effects in reducing inflammatory markers (COX-2, CRP and TNF-α) and enzymes involved in cartilage tissue degradation (MMPs and hyaluronidases), highlighting its therapeutic potential in modulating inflammation (Helli et al. 2019). However, the lack of significant reduction in IL-6 and IL-1β levels suggests that its anti-inflammatory effect may be selective or dependent on other factors. The effects of sesame may be mediated through alternative pathways and do not necessarily result in a direct reduction in IL-6 and IL-1β levels. The evaluation of additional biomarkers and further studies may provide a more comprehensive understanding of the effects of sesame in RA and its potential role as a complementary therapy. Studies investigating the effects of fish oil reveal a remarkable heterogeneity in their findings, underlining the complexity of its impact on RA treatment (Veselinovic et al. 2017; Dawczynski et al. 2018). In this regard, in one study, a significant decrease in the DAS-28 was observed in the experimental groups treated with fish oil, suggesting a beneficial effect in reducing RA disease activity (Veselinovic et al. 2017). However, in the other study, no significant improvement in DAS-28 was recorded, raising questions about the general efficacy of fish oil in all clinical settings (Dawczynski et al. 2018). This discrepancy was also observed in ESR and CRP measurements, with a significant decrease found in one of the studies (Veselinovic et al. 2017), while the other study reported no significant changes (Dawczynski et al. 2018). Furthermore, a significant decrease in the US-7 scale was recorded in one study, suggesting an improvement in disease activity assessment in that patient group (Dawczynski et al. 2018). These discrepancies may be due to various reasons, including differences in fish oil formulations, dosage, duration of treatment, or participant characteristics. Further research is needed to better understand the factors influencing the efficacy of fish oil as an adjunctive treatment for RA and to identify patient subgroups that may derive the greatest benefit from its use. Beyond efficacy outcomes, it is important to recognize that the pharmacokinetic properties and safety profiles of these nutraceuticals may influence their overall clinical impact. For instance, the limited oral bioavailability of lignans and CoQ10 may partly account for variability in outcomes, whereas omega-3 and primrose oils demonstrate consistent incorporation into plasma lipids and cell membranes, thereby sustaining their biological activity. Across the included trials, all interventions were well tolerated, with only occasional mild gastrointestinal symptoms reported, reinforcing their safety as adjunctive therapeutic options.

In another study, a significant decrease in TNF-α, a key proinflammatory cytokine in RA pathogenesis, was observed (Abdollahzad et al. 2015). However, no significant changes were observed in IL-6, indicating a potential specificity of CoQ10 in modulating certain inflammatory cytokines (Abdollahzad et al. 2015). Furthermore, a significant reduction in MDA levels, a marker of oxidative stress, was identified, whereas no significant changes in CAT were detected (Abdollahzad et al. 2015). These results suggest a possible role for CoQ10 in reducing oxidative stress and inflammation associated with RA, although further research is required to fully understand its mechanisms of action and clinical efficacy in treating this disease.

The results of studies investigating the effects of vitamin D in RA reveal diverse and challenging findings regarding its therapeutic efficacy (Buondonno et al. 2017; Soubrier et al. 2018). Despite the growing interest in vitamin D as a potential modulator of inflammation and disease activity in RA, the two reviewed RCTs did not show a statistically significant reduction of DAS-28 (Buondonno et al. 2017; Soubrier et al. 2018). This finding raises questions about the effectiveness of vitamin D supplementation in improving RA symptoms in the studied population. Regarding CRP, a marker of systemic inflammation, a significant decrease was observed in one RCT, suggesting a possible anti-inflammatory effect of vitamin D in some RA patients (Soubrier et al. 2018). However, the other study did not report significant changes in CRP, indicating a lack of consensus regarding the anti-inflammatory effects of vitamin D in this context (Buondonno et al. 2017). Cytokine assessment in one RCT revealed a significant increase in several cytokines, including TNF-α, IL-23, and IL-6, contradicting the hypothesis that vitamin D may exert anti-inflammatory effects through the suppression of proinflammatory cytokine production in RA (Buondonno et al. 2017). Considering the above, the complexity of vitamin D’s effects on the immune system and the need for a deeper understanding of its mechanisms of action in the context of RA must be highlighted. Finally, results from the HAQ functional disability assessment scale revealed a significant improvement in one of the studies, indicating a potential positive impact of vitamin D on physical function and health-related quality of life in RA patients (Soubrier et al. 2018).

Furthermore, regarding the current use of nutraceuticals, the European League Against Rheumatism (EULAR) has issued guidelines for the management of RA, which encompass the use of synthetic conventional DMARDs, glucocorticoids, biologic DMARDs, and targeted synthetic DMARDs. However, the EULAR recommendations do not explicitly address the use of nutraceuticals in the treatment of RA, therefore there is currently no clear indication for administration in the medical field (Smolen et al. 2020, 2023).

## Conclusion

This review underscores the diversity and complexity of nutraceutical-based therapeutic approaches explored for the management of RA. While several interventions demonstrated promising potential, inconsistencies across studies highlight the need for more rigorous investigation. The following key points emerge from the analysis:

- *Probiotics* consistently demonstrate potential in modulating systemic inflammation and disease activity in RA. However, the absence of significant improvements in catalase activity raises questions regarding their efficacy in mitigating oxidative stress, a critical component in RA pathogenesis.
- *Sesame supplementation* has shown beneficial effects on inflammatory markers and enzymes involved in cartilage degradation. However, its lack of effect on specific pro-inflammatory cytokines suggests a selective mechanism of action, possibly influenced by external or host-dependent factors.
- *Fish oil* studies revealed heterogeneity in clinical outcomes, underscoring the necessity for further trials to delineate the variables influencing its therapeutic response, including dosage, formulation, and patient characteristics.
- *CoQ10* showed favorable outcomes in reducing RA disease activity and oxidative stress-related biomarkers. Still, further research is necessary to substantiate these findings and elucidate the precise molecular pathways involved.
- *Vitamin D* trials yielded conflicting results regarding its immunomodulatory and clinical benefits. These findings reflect the complexity of its interaction with the immune system in RA and support the need for mechanistic studies and personalized therapeutic strategies.

Overall, the current evidence supports the potential utility of nutraceuticals as adjunctive therapies in RA. Additionally, the favorable safety profile observed across all interventions supports their potential role as adjunctive therapies in RA. Nevertheless, pharmacokinetic limitations, particularly poor bioavailability of certain compounds, remain a barrier to consistent efficacy and should be addressed in future trials through optimized formulations and standardized safety monitoring. However, robust, well-designed clinical trials are required to confirm their efficacy, determine optimal treatment protocols, and identify patient subpopulations most likely to benefit. Advancing this area of research may contribute to more personalized and integrative approaches in RA management, ultimately improving clinical outcomes and patient quality of life.

## Data Availability

Enquiries about data availability should be directed to the authors.

## Abbreviations

RA: Rheumatoid arthritis
TNF-α: Tumor necrosis factor-alpha
IL: Interleukin
NSAIDs: Non-steroidal anti-inflammatory drugs
DMARD: Disease-modifying antirheumatic drug
JAK: Janus kinase
PGE2: Prostaglandin E2
LTB4: Leukotriene B4
MMPs: Matrix metalloproteinases
NF-κB: Nuclear factor kappa-light-chain-enhancer of activated B cells
AP-1: Activator protein 1
IFN-γ: Interferon-gamma
SCFAs: Short-chain fatty acids
AMPK: AMP-activated protein kinase
SIRT1: Sirtuin 1
LDL: Low-density lipoprotein
ALA: Alpha-linolenic acid
EPA: Eicosapentaenoic acid
AA: Arachidonic acid
COX-2: Cyclooxygenase-2
RCTs: Randomized controlled trials
